# Naïve Theories of Emotions: Why People Might (Not) Be Uncertain or in Conflict About Felt Emotions

**DOI:** 10.5964/ejop.5529

**Published:** 2023-05-31

**Authors:** Vanda Lucia Zammuner

**Affiliations:** 1 Department of Developmental Psychology and Socialization Processes, University of Padova, Padua, Italy; 1 University of Bari Aldo Moro, Bari, Italy

**Keywords:** beliefs, conflict and uncertainty, felt emotions, emotion constellations, joy, pride, sadness, jealousy, envy

## Abstract

Beliefs about conflict and uncertainty over felt emotions—for Joy, Pride, Sadness, Jealousy and Envy events—were studied by means of Yes/No and Why questions. Each participant (N = 1,156) judged a typical antecedent for a single emotion—e.g., Jealousy: story protagonist SP sees his or her partner kiss someone. The Yes/No results showed that SP was frequently expected to experience both phenomena, the more so the greater the event impact (Yes range: 40–86%). Beliefs associated with Yes answers (BY) were categorized into 4 categories: (BY1) reason-emotion opposition—felt emotions are unreasonable, inadequate ways of reacting; (BY2) ambivalent emotions—e.g., joy and sadness; (BY3) unclear emotions; (BY4) other causes—e.g., focused on event implications, SP’s personality. No conflict or uncertainty answers (BN; range 14–60%) mirrored BY categories: (BN1) no reason-emotion opposition, (BN2) no ambivalent emotions, (BN3) clear emotions, (BN4) other causes. Attributions and beliefs about causes did not generally differ by gender. As a collective entity, expressed beliefs were complex, focusing on one or more emotion component—e.g., appraisal, regulation, expression—as well as on emotion intensity, duration, and on self-concept issues. Overall, expressed beliefs seemed to imply a malleability theory of emotions, and emotion awareness. Results overall confirmed the hypotheses that conflict and uncertainty attributions are more likely for: unpleasant experiences; when emotions are norm-incongruent for the judged event; when mixed, ambivalent emotions are felt. The study confirms that people interpret emotion processes according to their lay theories.

How people conceptualize themselves, their life and experiences, and the world in which they live in, i.e., what concepts or beliefs they develop and rely on to make sense of, and explain, their internal and external worlds are important issues, from a theoretical as well as a practical viewpoint. People’s lay theories—also referred to as folk, intuitive, implicit, commonsense, or naïve theories (e.g., Ford & Gross, 2019)—might be defined as comprising (sets of) beliefs (concepts, representations) about specific domains important to people’s life, about which people are concerned, i.e., ‘objects’ that define or affect the self and one’s social world.

This paper^1^1See Notes and Appendix in Zammuner (2023) for additional information., reporting the integrated, mainly qualitative results of five parallel studies, explores whether people believe that conflict and uncertainty, relevant aspects of emotion experiences, might be associated with felt emotions, and why (their causes, explanations). To set the theoretical background for the present study questions, the next section briefly discusses what is meant by lay theories and why they are studied, providing a few examples of studied ‘objects’.

## The Study of Beliefs About Emotions

As argued within social cognition approaches (e.g., Molden & Dweck, 2006), beliefs about *object x* are likely to motivate and guide behavior. Lay theories have thus been studied extensively in psychology, studying the impact of such beliefs and related motives and behaviors in relation to a variety of objects (for recent partial reviews, see De Castella et al., 2018; Ong et al., 2015; Tamir et al., 2007)—e.g., beliefs on the nature (functioning, causes, effects, etc.) of intelligence, morality and personality; beliefs about such issues as altruism (Carlson & Zaki, 2018), suicide causes (Voracek et al., 2007), and compulsive behaviors in eating-disorder patients (Lawson et al., 2007). Obtained results overall support the hypothesis that people *do entertain* beliefs, often culture-specific ones, on subjectively relevant objects, and that such beliefs contribute to influence their thinking, attitudes and behaviors.

A *specific object* (domain) of many belief studies in the last five decades is *emotion*, paralleling increased attention to its study (Barrett et al., 2016). A compilation of research on emotion beliefs is still lacking. We might however argue that many studies on such constructs as concepts, prototypes, scripts and stereotypes did actually explore lay beliefs (Zammuner, 2023, N1). Over time, studies increasingly focused on beliefs about *aspects (components) of emotions*, such as expression, appraisal, and regulation. For instance, according to recent studies (e.g., Ford & Gross, 2019; Tamir et al., 2007) people are more likely to regulate their emotions if they hold a *malleability* rather than a *stability* theory, i.e., if they believe that emotions can change rather than be immutable.

When discussing emotion beliefs, it is important to highlight their cultural basis, considering attitudes (standards) toward emotions and their expression, i.e., what standards culture encourages (e.g., whether and how anger should be expressed), and how standards change in personal history, time and socio-cultural context—e.g., in child-rearing affection-norms (Shields & Koster, 1989; Stearns, 2019), or in collectivist *versus* individualist cultures (Eid & Diener, 2001). The results of such studies confirm that emotional experiences show systematic cross-cultural differences (in addition to similarities) mutually constitutive with culturally-based practices and *meanings* (i.e., the beliefs; Mesquita & Leu, 2007).

In discussing emotion beliefs, finally, we need to consider self-concepts, self-construals, as they influence emotion experiences and behaviors (Leary, 2007; Markus & Kitayama, 1991)—e.g., an incoherent self-concept predicts lower psychological well-being (Diehl & Hay, 2011), whereas a fit between actual- and ideal-self-view influences positive affect and self-esteem (Pelham & Swann, 1989). More generally, self-related motives, such as enhancement and verification, are likely to influence people’s goals when experiencing and expressing an emotion—e.g., when judging *emotion_e_* appropriateness *in context_c_*, or its expression.

### Lay Emotion Theories as Influences on Emotion Experience and Its Judgement

To discuss emotion beliefs, we need to recall, first, that emotions are *multi-componential processes* (Scherer, 1984)—e.g., comprising the experience of un/pleasantness, physiological and expressive responses, appraisals and action tendencies. A further component is *significance* (Frijda, 1986): people reflect on and judge their emotions, as un/controllable, un/desirable or un/acceptable. Significance triggers, and reflects, emotion regulation. Second, emotions are best conceptualized as *episodes* (Frijda, 1986): response sequences during which several emotions, of varying intensity and duration, might be felt, in close succession (Grossmann et al., 2016; Sonnemans & Frijda, 1994). Third, most emotions are *interpersonal phenomena*, dealing with, and relevant for, subjectively important interpersonal transactions. Many relationship features—e.g., how a relationship develops—are defined by felt emotions and actions, including facial and bodily expression (e.g., Omarzu, 2000). Fourth, social relationships transactions occur within contexts characterized by (sets of) features—e.g., cultural norms, a person’s role in the eliciting event—whose values may interact in their impact. People’s beliefs about context-relevant features are likely to shape their emotions, behaviors and evaluations.

Emotion beliefs focus on various aspects of emotions (e.g., whether they ought to be regulated, expressed, etc.) and, as such, constitute people’s *lay theories of emotion (LTE)*. According to Zammuner (2000), LTE include beliefs of different kinds, namely, *descriptive* (e.g., ‘*sadness is an unpleasant emotion*’) and *prescriptive ones* (*e.g., ‘don’t show your anger’*). Furthermore, beliefs are *emotion-specific* when they focus on category members (sadness, joy, envy, etc.), and *emotion a-specific* when focusing on the superordinate emotion category (e.g., ‘*intense emotions leak through’*). Moreover, *beliefs* might be *context-free, i.e*., believed to be true independently of the context in which the emotion occurs (e.g., ‘*always count to ten before showing your anger*’), or be *context-bound (e.g., ‘it’s ok to express some anger to your husband when you* are *arguing with him’)*. Most beliefs are likely to be congruent with culturally based ones as regards the meaning, adequacy, and legitimacy of emotions (in general, and for specific emotions), on how to express them, and so forth (e.g., Johnson-Laird & Oatley, 2000; Mesquita & Leu, 2007; Russell, 1991). Socio-demographic variables such as gender, religion, or age (e.g., adolescent *versus* adult) are likely to influence beliefs—e.g., adolescents’ gendered emotion beliefs might differ to some extent from adults’ ones; which beliefs are culturally salient might thus vary across contexts, as a function of salient variables—e.g., person *P*’s gender or age.

Finally, emotion beliefs are likely to constitute rich, salient *knowledge structures* because emotional experiences are pervasive and, given their adaptive functions, attended to with much attention (e.g., Keltner & Gross, 1999). Indeed, emotional complexity (e.g., experiencing multiple emotions in relation to a single event; Grossmann et al., 2016; Lindquist & Barrett, 2008) might index emotion-knowledge richness.

In sum, people’s lay theories of emotions, comprising a variety of beliefs, are expected to influence people’s (unconscious or *vice versa* intentional) perception and evaluation of their own (and others’) emotion experience—in terms of its adequacy, norm-congruency, and in relation to inter/personal goals at stake and relevant contextual features, influencing how the person perceives her emotion experience, how it develops in time, and if and how emotion regulation will occur.

## Conflict and Uncertainty Over Felt Emotions

### Research Aim, Overall Design and Hypotheses

Although people’s knowledge of emotions has been much studied, we still do not have a full understanding of it. The aim of the present study—part of a larger research project on lay theories (Zammuner, 2023, N2)—was to assess whether people believe that emotion experiences are associated with conflict and uncertainty, and why they think so. A related aim was to test whether beliefs differed according to the *nature of eliciting events* and *felt emotions*, as specified below.

To address these theoretical questions, the study focused on *typical* events eliciting *emotion constellations*, i.e., emotion-type structures, defined as emotion episodes by Frijda (1986), that might comprise *several* feelings, cognitive, physiological, expressive and behavioral responses. In other words, an event might elicit more than one emotion (each of which might be associated with cognitive, physiological, etc. responses). The studied constellations were JOY, PRIDE, SADNESS, JEALOUSY, and ENVY—indicated by capital letters in this paper; specific emotions that might be felt or expressed *within a constellation* or *across them* are instead indicated by lower case characters (e.g., jealousy, sadness, joy). Constellations differed by their: (i) overall *Valence,* i.e., pleasant versus unpleasant, and (ii) *Normativity* of emotions, i.e., extent to which felt emotions are norm-congruent. Further, constellation-specific events differed by their typical *salience*.

These theoretical questions were operationalized in a paper-and-pencil questionnaire (see the Method section). Participants focused on the emotional experience elicited by an event narrated by its *story protagonist SP*, and answered *two* (open and closed) *question couplets* (Zammuner, 2023, N2) (vignette and immediate-recall methods can obtain very similar emotion reports; Robinson & Clore, 2001). Table 1 reports the judged events gist, and a partial list of constellation-specific emotions participants could attribute to SP.

**Table 1:** Events Occurring to Story Protagonist SP, Listed Constellation-Specific Emotions and Frequencies of Yes-Answers

			Yes answers (%)	
Constellation event	Event gist	Listed constellation-specific Emotions	C	U
JOY S—Lottery	SP wins a big amount of money at a lottery.	euphoria, hope, cheerfulness, relief,	48	60
JOY L—Trip	SP spends a very pleasant day with friends on the beach.	glad, serene, guilty, uneasiness	55	49
PRIDE S—Job	SP is selected, after an interview, for a job at a high-ranking firm.	triumph, self-satisfaction, excitement, pride,	51	55
PRIDE L—Partner	SP’s friends approve of, and cheer her new sentimental partner.	worry, satisfaction, embarrassment	40	45
SADNESS S—Death of close person	SP is told that someone (friend; grandfather) died	nostalgia, pain, impotence, resignation, shock, melancholy,	63	73
SADNESS L—Death of acquaintance/dog	SP’s dog died; A SP’s acquaintance died.	apathy, guilt, agitation, anguish, depression	59	57
JEALOUSY S—Kiss	SP sees partner passionately kissing someone.	jealousy, envy, anger, frustration, disgust,	86	68
JEALOUSY L—Flirt	SP sees her partner devoting attention to someone else.	irritation, depression, insecurity	84	64
ENVY—SP’s colleague C is promoted; C has:	1. S—Less capacity;	jealousy, envy, hurt pride, contempt, vengeance,	70	52
	2. S—Equal capacity;	hostility, resentment, humiliation, indignation,	85	68
	3. L—Greater capacity.	irritation, insecurity, depression	85	72

*Note.* S = Serious, L = Light. C = Conflict, U = Uncertainty. Values for Yes answers are percentages within event.

### Hypotheses

Both conflict and uncertainty might theoretically occur, and be relatively frequent, in relation to emotion experiences if we assume that a person *P* evaluates to what extent her experience elicited by a salient event is congruent with her goals and concerns in the event context, and with individually-salient emotion norms. Conflict and uncertainty might therefore characterize such evaluations—e.g., about the contextual appropriateness of a feeling, or an appraisal, or how to re-act toward the event causal agent. The literature on both *interpersonal* and *intrapersonal* conflicts (Zammuner, 2023, N3) converge in stressing the importance of emotion-related processes and skills (including self-understanding and awareness, regulation and coping strategies) in defining the extent to which people will experience conflict, and deal adequately with its origins and its short- or long-term effects. Both conflict and uncertainty are unpleasant cognitive and emotional states. People might thus try and avoid them, and/or try and get out of them once felt, as illustrated by the ‘classic’ love-hate ambivalence. To get rid of conflict and/or uncertainty, people might ask themselves about ‘causes of’, and ‘solutions for’, the events that elicited such unpleasant states. If, as discussed above, people’s knowledge of emotions is rich, including beliefs about typical experiences (e.g., of SADNESS) and context-salient emotion-norms, then conflict and uncertainty attributions might be triggered by beliefs that focus on this or that aspect of the experience—including hedonic tone of emotions, event appraisal, cause or source of emotion(s), relevant norms and personal goals in relation to the event, self-concept, present or future event implications for the self and/or for event-salient relationships, and on regulation issues (e.g., whether to regulate an emotion and how, depending on known regulation strategies). The mentioned differences between constellations, and constellation-specific events, were expected to influence conflict and uncertainty attributions.

Feeling *in conflict* over felt emotions presupposes that person *P* has some awareness of her emotions. *Awareness* might be based on perceptual salience (Frijda & Zammuner, 1992), i.e., what ‘material’ (e.g., the event implications for the future) *P* most easily focuses upon to meet her goals and contextual constraints. Felt conflict might thus trigger (beliefs about) *emotion regulation*, affecting experience and/or expression. P’s beliefs are expected to focus on intra- and/or inter-personal consequences of felt emotions.

*Uncertainty* over felt emotions might be construed as poor emotion awareness and understanding—in the literature, *awareness* refers to both the *attention* paid to emotions, and to the *clarity* about which emotions are felt, whereas *understanding* refers to the capacity to differentiate emotions one from the other, to know their causes, etc. (Boden & Berenbaum, 2011; Diehl & Hay, 2011; Lindquist & Barrett, 2008). However, as feeling mixed, complex emotions can be interpreted as an emotional-complexity index (Lindquist & Barrett, 2008), equating uncertainty with low awareness and understanding might be misleading. An alternative hypothesis, underlying this study, is that uncertainty—though it might at times imply low emotion awareness and understanding—is caused by *complex-experience* attributions, i.e., attributions of feelings that are concomitant, and/or unclearly perceived, and/or implying contrasting features, e.g., in terms of event appraisals, or implications for the protagonist’s (present or future) goals and related behavior. Uncertainty might trigger conflict over felt emotions and their implications (but not necessarily so), whereas by itself it might not necessarily trigger conflict.

Four *general* hypotheses underlie the study. *Conflict* (C) and/or *Uncertainty* (U) attributions are expected to be *more frequent*: (H1-CU) when the experience is overall unpleasant rather than pleasant, due to the greater attention paid to it because of its salience, and the need to restore a positive state; (H2-CU) for ‘serious’ than ‘light’ events, due to the greater salience of the former; (H3-CU) if specific felt *emotions, and/or their expression,* are perceived as norm-incongruent, from a personal and/or social viewpoint, in relation to salient contextual features. That is, given the circumstances—the event (see Tables 1 and 2)—specific emotions might generally be judged as adequate, appropriate, legitimate with respect to personal and/or social standards (e.g., sadness for SADNESS, joy for JOY), or *vice versa* as either generally inappropriate, or as norm-congruent for event E1, but incongruent for event E2 (e.g., pride for PRIDE-light event; contempt or indignation for ENVY-equal-capacity event; depression or anger for JEALOUSY-flirt); or, finally, emotions might be judged as subjectively legitimate although norm-incongruent. If normative beliefs are activated, need-for-regulation beliefs might become relevant: person *P* might solve a felt conflict by *regulating* her experience and behavior according to norms—e.g., if *P* feels like crying because her dog died, but believes others would judge it inappropriate for such a loss, she might suppress her action tendency. Note, however, that what counts as a relevant contextual norm might be subjectively unclear, because norms are subtle or complex—e.g., *context-specific norms* might vary according to gender—or, *individually salient* context-specific norms might clash with socially accepted ones; (H4-CU) if the experience includes concomitant emotions (e.g., jealousy, anger, insecurity; joy and pride), and/or ambivalent (or contrasting) ones (e.g., relief and sadness, joy and envy). In such cases conflict attributions are triggered by multiple and/or contrasting event appraisals (of its meaning, relevance, etc.), action tendencies and/or actual behaviors. Conflict in turn might induce uncertainty on the most appropriate or salient event-appraisal, and/or its ‘correctness’, perhaps inducing a re-appraisal of it, or about the most desirable course of action. Both H3-CU and H4-CU presuppose emotion awareness, i.e., self- and emotion-understanding; conflict and/or uncertainty should not occur if such capabilities are absent.

**Table 2:** Attributed Conflict and Uncertainty (Yes Answers) to Felt Emotions and Types of Cause Within Emotion-Constellations

	Constellation									
	JOY		PRIDE		SADNESS		JEALOUSY		ENVY	
Variable	C	U	C	U	C	U	C	U	C	U
Percentage of participants who answered *Yes^a^*	52	55	45	56	61	65	85	66	80	64
Type of cause^b^									
Reason vs. emotion	16	10	26	9	43	12	38	29	48	24
Contrasting or unclear emotions	58	76	66	84	41	65	25	59	28	39
Other causes	26	14	8	24	16	23	38	12	24	37
Total (over supplied *Yes* causes)	100	100	100	100	100	100	100	100	100	100

*Note.* C = Conflict, U = Uncertainty. The table shows percentage frequencies, averaged over constellation-events.

^a^For ENVY and SADNESS, the frequency of *Yes* versus *No* answers for Conflict and/or Uncertainty varied significantly according to event: Conflict ENVY: χ^2^(2) = 5.62, *p* < .06; Uncertainty ENVY: χ^2^(2) = 5.97, *p* < .05; Uncertainty SADNESS: χ^2^(1) = 6.57, *p* < .01. ^b^Type-of-cause frequencies were computed on total number of causes supplied within each constellation.

More *specific* hypotheses regard *Uncertainty*, expected to be *more frequent* when: (H5-U1) the experience is not easily, clearly perceived because it is either diffused, of low intensity, or *vice versa* is too intense; (H6-U2) the experience is complex, i.e., includes concomitant emotions, making difficult to grasp their cause, implications, etc.; more specifically, differentiating emotions and/or understanding their cause is difficult because the emotions hedonic tone and nature (H6-U2.1) is similar (e.g. sadness and depression), or (H6-U2.2) is opposite (e.g., joy and sadness, love and hate), associated with contrasting implications (this is actually a sub-hypothesis of H4-CU); (H7-U3) the person does not know if and how subjectively undesired and/or norm-incongruent felt emotions should be regulated (repressed, attenuated, etc.).

As the collected data in this study are mostly of a *qualitative nature* (participants’ answers to the *Why* questions), hypothesis (dis)confirmation can be only at the qualitative level.

## Method

### Participants

Casual samples of Italian men (*N* = 546) and women (*N* = 610), all university students (age range: 18–33 years, range mean-age: 23–24 years) participated in five parallel studies, i.e., conducted at about the same period, using a questionnaire whose structure was identical (see below the ‘Experimental Stimuli and Procedure’ Section); Joy: *N* = 240, Pride: *N* = 195, Sadness: *N* = 240, Jealousy: *N* = 301, Envy: *N* = 180. Participants were recruited at several classes in various faculties in Northern Italy. Participation was voluntary. Participants who had not answered conflict and uncertainty questions were excluded from this study.

### Experimental Stimuli and Procedure

Each of the five constellations was assessed by *two or more events* (see Table 1) differing in ‘*seriousness’ (salience)* and likely degree of cultural or personal *norm-congruency*, legitimacy. More specifically, in comparison to ‘*light’* events, describing less self-relevant situations, ‘*serious’* events were expected to elicit *more intense* experiences due to their salience for personal concerns, and to elicit emotions more likely to be judged in terms of their *norm-congruency* given the event implications for legitimate concerns. To exemplify, norms justify feeling very sad for an important loss, or happy in satisfying a personal goal, but might not justify anger or jealousy about one’s partner’s brief interest in someone else. In other words, beliefs activated by participants as relevant ones were expected to vary according to goals, concerns, and norms characterizing a specific-event experience. Differences between event-elicited experiences were expected to help our understanding of beliefs as regards both felt emotions and their context-defined norm-congruency.

Each participant answered a questionnaire describing a *single event*, narrated by its *story protagonist SP*—e.g. for JEALOUSY, SP sees his or her partner kiss someone (see Table 1). Participants made *prototypical* attributions, i.e., were asked to describe “how *in general* the protagonist of an event like this would feel and act”. To maximize identification with SP, each participant judged a version with a same-sex SP. After answering other questions about SP’s emotional experience (not discussed in this paper; see Zammuner, 2023, N2 and its references), participants answered two question couplets: “Do you think the protagonist might feel: (i) in conflict, (ii) uncertain about his or her felt emotions (*closed* question, with Yes/No answers), and “Why?” (*open* question).

#### Overall Experimental Design, and Questionnaire Versions

Several questionnaire versions were created that differed on the following variables. *(a) Constellation*, each reporting one of two or more typical events. (b) *Event type*, expected to differ in subjective salience and norm-congruency of elicited emotions (Table 1). For instance, SADNESS participants judged one of 4 loss events, two ‘serious’ ones (death of one’s own grand-father or of a close friend) and two ‘lighter’ ones (death of an acquaintance, or of one’s own dog). ENVY participants judged instead a single event type—SP’s colleague C is promoted to a higher position—that across versions differed in its justifiableness perception. (c) *Listed emotions*—selected from literature theoretical and empirical analyses—which participants rated as felt ones (see Table 1). Joy, surprise, fear, anger and sadness were always included; a few listed emotions served as control stimuli—e.g., PRIDE and JOY events were *not* expected to elicit sadness or fear. (d) *Sharing interlocutor SI* (*...SP “wants, or happens to talk with (SI) about his (her) event experience…*”). SI varied in terms of: (i) affective closeness, and (ii) causal involvement or agency (yes or no) in the event; agency was salient for ENVY (when SI was SP’s colleague rather than the partner), and JEALOUSY (when SI was SP’s Partner rather than a friend). Finally, (e) *SP sex*, matching participants’ sex. In sum, *Constellation*, *Event type* and *Sharing interlocutor* were between-subject variables; *Listed emotions* were within subject-variables.

### Data Analysis

Participants’ *Yes* or *No* answers to the two conflict and uncertainty closed questions were coded numerically, whereas answers to the two open questions were content-analyzed, using the procedure described next.

#### Qualitative Analysis of Beliefs: Content Analysis

Participants’ answers to the two open questions (see Tables A1–A4 in the Supplementary Materials for literal quotes) were content-analyzed within each emotion-constellation using a theoretically inspired data-driven method that produced, at the various coding phases, analytical coding categories; the final schema was a list of higher-order categories coding participants’ constellation-specific beliefs (Zammuner, 2023, N4). Raw frequency scores (0 to maximum 3 for each category) were assigned to each participant (the data included specification of judged event, and participant’s socio-demographic characteristics); total frequencies were computed separately for each event. The inter-coder within-constellation agreement index (Krippendorff’s *alpha*) obtained by comparing independently assigned author- and judge-codes, computed, regardless of judged event, on a random 10% protocol sample, was acceptable, with individual category agreement above 75% (range 78%–90%).

To compare belief-type *frequency* across constellations, original within-constellation categories were re-coded or re-grouped (rephrasing them if necessary) by the author on the basis of their conceptual similarity—to adequately represent the meaning of each original category, with its component sub-categories, into the present-study *across-constellation* categories (see Table 2), literal quotes of constellation-specific categories were carefully looked at whenever necessary. In sum, to compare beliefs across constellations interesting specificities had to be disregarded. The Results section will however report some information on constellation- and event-specificity of beliefs.

#### Quantitative Analyses

Frequencies of *Yes* and *No* answers to Conflict and Uncertainty closed-questions, and raw frequencies of the three main belief categories that emerged in this study were analyzed with the χ^2^ statistics to check differences by event type within constellation (Zammuner, 2023, N2) (Tables 1 and 2).

## Results

Table 1 reports *Yes-*answer frequencies of conflict and uncertainty attributions over emotions to the story protagonist SP, subdivided by event type and constellation; *No*-answer frequencies can be inferred from them. Table 2 reports frequencies of belief motives for which SP might feel in conflict or uncertain (*Yes* answers, disregarding event differences). Such attributions and their motives are perhaps best understood considering that participants often believed (Zammuner, 2023, N2) that felt emotions would not be shared sincerely, i.e., would be regulated according to norms, by attenuating their intensity, or by not talking about them at all, or finally by reporting felt emotions with a greater intensity than actually felt (as reported, for instance, in Zammuner, 2011). Such discrepancies are likely to represent ‘ground data’ for participants’ attribution of conflict and uncertainty.

### Beliefs About Conflict and Uncertainty: Yes/No Attributions

*Yes*-answer frequencies to the two questions “Do you think the protagonist might feel in conflict”, “…uncertain about his or her felt emotions?” (Tables 1 and 2) show that both phenomena were frequently attributed to story protagonist SP—range across constellations and events: 40—86%; mean frequency: Conflict 66%, Uncertainty 60%. Conversely, *No*-answers ranged from 14% to 60%. These results suggest that for many participants felt emotions are not a fixed entity, something that one just takes for given, cannot do anything about, i.e., participants hold a *malleability* view about emotions (Ford & Gross, 2019). If participants held the opposite *entity* theory, they would have replied *No* to either question. Indeed, as reported below (see the 'Beliefs about Conflict and/or Uncertainty Absence’ section), even when their reply was *No* Conflict and/or Uncertainty, participants’ accounts on the whole did not imply entity beliefs.

As hypothesized (H1-CU), both conflict and uncertainty were attributed (Table 2) more often to SADNESS, JEALOUSY and ENVY, negative constellations (range: 60 to 85%) than to the positive ones (45 to 56%). Within negative constellations, conflict was typically much more frequent than uncertainty (JEALOUSY, and ENVY) or about as frequent (SADNESS). Within the positive constellations JOY and PRIDE (except for JOY-Light event) uncertainty was instead slightly more frequent than conflict, a result better understood by considering its specific causes, as discussed below.

Within constellations, *event type* further differentiated attributions, with both phenomena characterizing *serious* events (except ENVY) more than *light* ones, overall supporting H2-CU—e.g., PRIDE participants attributed both to the serious-event protagonist in about 10% more of the cases with respect to the light one (see Table 1).

Conflict and uncertainty attributions are better understood with reference to the normative status of event-elicited emotions (see Table 1). To illustrate, the ENVY ‘Promotion of a less capable colleague’ event elicited the least conflict, and especially the least uncertainty (Table 1): participants in fact expected SP to feel intense anger, irritation, resentment, hostility, humiliation, indignation, and contempt, i.e., emotions (much less relevant for the other ENVY events) implying that SP appraises the event as ‘unjust’, and expect others to appraise it similarly. Because the (attributed) emotions are judged as legitimate, SP is believed to feel less or no conflict at all over them, and less uncertainty. The results, more generally, showed that participants were more likely to expect conflict when emotions are judged to be norm-incongruent, supporting H3-CU. For instance, conflict was, overall, much less frequent for SADNESS, i.e. when legitimate emotions such as sadness, pain, shock, are felt, than for JEALOUSY and ENVY, constellations typically comprising negatively sanctioned emotions such as jealousy and envy (Zammuner, 2023, N2). Likewise, conflict was not totally absent even for the positive constellations of PRIDE and JOY, a result, according to participants' beliefs (see sections below and Tables A1–A4 in the Supplementary Materials), related to the norm-incongruent nature of some felt emotions, such as pride for PRIDE, and Euphoria for JOY. Uncertainty was the most frequent for the SADNESS event causing SP to suffer a very salient loss, and for the two ENVY events in which the colleague promotion is not really unjustified, events that elicited frequent attributions of conflict too.

### Beliefs About the Causes of Conflict and Uncertainty

This section reports how participants explained conflict and uncertainty presence (subsections, ‘Reason Versus Emotion Discrepancy’–‘‘Other’ Causes That Explain Felt Conflict and/or Uncertainty’) or absence (subsection ‘Beliefs about Conflict and/or Uncertainty Absence’), i.e., what their beliefs focused on—e.g., feelings, or specific emotion components such as appraisal—allowing us to better understand their attributions.

Three main *high-order* categories resulted from analyzing all beliefs associated with *Yes* conflict and uncertainty answers (see the Method section, and Table 2). Namely, *(a) felt emotions are at variance with reason*, i.e., with rational (shared or personal) norms about what one should feel; (b) *feeling contrasting and/or unclear emotions*—grouped together because both belief types (b1) explicitly focus on emotions, and were often (b2) mentioned together in participants’ accounts, and/or originally coded within the same category (contrasting and unclear causes, especially the former, often implied a ‘reason-emotion opposition’); (c) ‘*other causes’*. Participants’ beliefs associated with *No* answers, denying conflict and uncertainty, were coded by similar high-order categories (see the subsection ‘Beliefs about Conflict and/or Uncertainty Absence’). Such abstract, summary-like semantic categories do not give justice to participants’ variety, subtlety and complexity of conceptions—e.g., many categories varied significantly in their frequency according to event, e.g., to which emotions it elicited, and why. Such belief variety and complexity is however well illustrated by participants’ literal quotes (*Q*), many of which are reported in the Supplementary Materials, Tables A1–A4.

#### Reason Versus Emotion Discrepancy

Beliefs in the Reason–Emotion discrepancy category focus on asking oneself whether felt emotions are at variance with rationality, i.e., with personal or culturally shared norms about what one should feel, and why. In other words, beliefs focus on evaluations of what ideally one should feel, what would be best given the circumstances (i.e., the eliciting event), taking however also into account one’s own ideal self-concept, goals and expectations (see Table A1 quotes in the Supplementary Materials). The ‘Reason-Emotion’ discrepancy was a rather frequent cause of conflict (Table 2), especially for SADNESS, JEALOUSY and ENVY (about 40 to 50% of participants); less frequently, it was however invoked for PRIDE and JOY conflict too. It was not infrequent (range 9–29%) as a cause of uncertainty too, relevant especially for JEALOUSY and ENVY (see in the Supplementary Materials, Table A1 quotes Q4, Q7, Q15, Q16, Q26).

The kind of norms called upon by participants (i.e., which felt emotions are believed to be potentially norm-incongruent) are quite varied, focusing on emotion-related components and features. More specifically, norms were related to: (i) *emotion intensity*, e.g., judged too weak in comparison to expectations (Q3–Q4); (ii) idealized or desired aspects of both private and public self-concept, focusing on whether emotions might clash with *personally salient norms* (Q8–Q11) that call into question one’s values and personality (Q5)—e.g., is sorrow for the loss sufficiently intense to reflect one’s love capacity?—or *social ones* (Q7)—e.g., are envy and hurt pride acceptable when a colleague is promoted?—or *personal goals* in the specific context (Q12)—e.g., not wanting to appear as someone blinded by anger; (iii) event appraisal, e.g., questioning its ethical value in relation to self (Q17–Q20), or, more generally, (iv) *present, past or future event implications* in relation to its appraisal (Q21–Q26), with beliefs focusing on implications for personal values, or possibly conflicting evaluations by the self and one’s friends, or the contrast between present and future feelings, or even (Q25) asking oneself whether one’s past behavior toward the event agent caused the event, an appraisal that could induce seeing it as justified (appraisal change), thus modifying the event implications for the future. In some cases, participants focused on (v) *action tendencies* (Q11, Q12, Q14, Q26), and (vi) *coping strategies* for the event. Furthermore, many accounts *implicitly* referred to (vii) *regulation* (of feelings and their intensity, action tendency, etc.), focusing on achieving (viii) *short- and long-term concerns and goals*, e.g., were related to optimal strategies for managing SP’s relationship with individuals who played a salient role in the event (e.g., promoted colleague; unfaithful partner), or were the sharing friend. In sum, the Reason–Emotion beliefs—focused on emotion features (intensity, appraisal, agency, etc.) and ‘rational’ causes (e.g., norms)—often implied awareness of emotions, their causes and implications. Overall, beliefs in this category support the hypotheses about norm-incongruity (H3-CU), emotion intensity (H5-U1) and ambivalence (H6-U2.2), and regulation issues (H7-U3).

#### Feeling Concomitant and Ambivalent, Contrasting Emotions

Participants’ attributions frequently focused on the belief that the experience is complex, due to concomitant and/or especially ambivalent, contrasting emotions—e.g., joy–worry, joy–guilt, pride–anxiety, happiness–sadness, anger–love (see Table A2 in the Supplementary Materials). Beliefs in this category—about 20% more frequent for Uncertainty than Conflict attributions (Table 2), often focused on emotion *causes* and *implications*, rather than, or in addition to, the feelings themselves (as was the case for the Reason–Emotion category). For instance, participants believe that contrast might arise from a present-past clash, i.e., what one feels now towards the emotional object is opposite to what one used to feel (Q31–33)—e.g., *An event cannot alter radically in a brief time what you felt toward a person*. Contrasting emotions might induce a perspective shift in appraisal (Q40–41, Q44)—e.g., *If she puts herself in her boy-friend’s shoes, she cannot condemn totally his behavior*—and/or reflect opposite action tendencies (Q38–39, Q44–45, Q49–50)—e.g., (SP) *is in despair, but (...) one wants to stop suffering*; (conflict between) *getting another animal* (and) *being faithful to its memory*. Implicit in, or related to, action-tendency evaluations are beliefs focusing on best ways to cope with the event, and implications for behavioral choices (Q46–47)—e.g., *wanting to avoid thinking about the situation, versus facing it completely*. Opposite action orientations might also result from a potential clash between private and public self—*she wants to be reassured and consoled, instead she behaves as if untouched by the event; wants to be strong and behaves aggressively, but in her heart she only wants to cry*. Contrasting appraisal and action tendencies are associated with different event implications, as a function of focused-upon aspects; e.g., to what extent the event satisfies a concern (Q30, Q42), a goal (Q43), an expectation (Q53), or as regards its present or future implications at (Q51–52)—e.g., *afraid of the changes it could bring in her life*. In sum, in participants’ beliefs, feeling concomitant and especially ambivalent emotions (and appraisals, action tendencies, etc.) means the person is torn between different ways of reacting to the event, a fact in itself unpleasant, confusing. To solve conflict/uncertainty, SP is believed to question her experience’s congruity with rationality, norms, her goals and concern, a questioning that in turn implies SP’s attempts at regulating emotions (appraisals, action tendencies, etc.) to make them normative or subjectively more acceptable—e.g., repressing an emotion and/or intensifying it; implementing the most appropriate action tendency. Beliefs in this category support hypotheses H4-CU on concomitant and ambivalent emotions, and H6-U2 on emotion complexity.

#### Feeling Unclear Emotions

Participants believed that the presence of unclear emotions explain both phenomena, but uncertainty more frequently than conflict. Although some simply stated “*SP feels unclear emotions*”, many participants expressed their beliefs more precisely (see Table A3 in the Supplementary Materials). For instance, SP is uncertain about what she *does* feel (Q54); the event-caused excitement makes emotional awareness difficult (Q55); SP feels concomitant emotions (Q56, Q58, Q61, Q64, Q69, Q70, Q72–Q74); SP has difficulty differentiating emotions of a similar hedonic tone but with a different intensity, or with a too similar intensity (Q59, Q71). In this category too, beliefs often focused on specific experience features, components or dimensions believed to reduce emotion clarity (Table A3 in the Supplementary Materials), including: intensity of emotions, either too strong or weak (Q61–Q65, Q69–Q71); high activation, inhibiting a reflexive stance (Q55, Q59, Q62) and causing loss of rational control (Q58, Q70); (brief) duration of emotions, implicitly referring to emotion intensity and/or activation level (Q65). As causes of concomitant or intense emotions many beliefs focused on appraisal, mentioning event’s novelty, unexpectedness (Q79–Q82), high salience (Q77–Q82), implications (Q83–Q87), and SP’s difficulty to decide the ‘correct way’ to appraise the event (Q76, Q78) and deal with it, immediately or in the future (Q74, Q83–Q87). Beliefs at times were more general: being in an emotional state implies inability to accurately perceive reality, including the internal one (Q58, Q63, Q66, Q67, Q70), i.e., lack of clarity is a distinct feature of emotional experiences—a quite old concept in western lay thinking. Other general-nature beliefs focused on the complexity of experiences—e.g., ... *emotions and feelings … have 1000 nuances*... (Q60)—and the difficulty of labeling them—*It’s completely normal to feel uncertain because there are more emotions than words that designate them* (Q68). In some cases, uncertainty, similarly to conflict, was attributed to not knowing whether one is conforming to the norms (Q57). Finally, several participants explained ‘unclear’ emotions as due to concomitant causes—e.g., feeling “*concomitant and incoherent emotions*” …norm incongruity as an additional cause of ‘confusion’, and *intentional* unawareness to protect the self-image (Q15, Table A1 in the Supplementary Materials). In sum, Unclear-emotion beliefs focused on various experience aspects, especially concomitant, contradictory, and at times too strong or too weak feeling states, and on uncertainties about event appraisal, highlighting, on the whole, experience complexity. Overall, beliefs in this category contribute to support hypotheses about emotion complexity (H6-U2), ambivalence (H6-U2.2), intensity (H5-U1), and regulation issues (H7-U3) about felt emotions.

#### ‘Other’ Causes That Explain Felt Conflict and/or Uncertainty

Participants focused on ‘Other causes’ too (Zammuner, 2023, N4) (Table 2). Their frequency was quite low (range 8–16%) for PRIDE and SADNESS Conflict, and for JEALOUSY and JOY Uncertainty, not so low in the remaining cases (23–38%). Many ‘Other’ beliefs referred to event-specific present or future implications for SP, especially for SADNESS and JEALOUSY, e.g., mentioning SP’s fears about the relationship future in JEALOUSY (Q88–89; Table A4 in the Supplementary Materials), or SP’s future emotions or thoughts following the loss in SADNESS (Q90), or the event implications for SP’s self-esteem when the equal-ability colleague is promoted (Q91). For all constellations, conflict or uncertainty Other causes included focusing on SP’s personality (Q92), or, more generically, how the event fits into its context, and/or its interpretation according to SP’s mood or personal characteristics (Q93), stressing a relativistic, idiosyncratic, ‘it depends from’ approach. Finally, some participants focused on sheer experience-complexity (Q95), its opaqueness to rationality (Q94), or on human psychology complexity (Q96).

#### Beliefs About Conflict and/or Uncertainty Absence

As expected, not all participants believed that one feels conflict or uncertainty: 14% to 60% across constellations and events in fact gave *No* answers (Table 1). Specifically, whereas only a minority believed that SP in JEALOUSY and ENVY would feel neither (frequencies varied with the event; *No* conflict range: 15–30%, *No* uncertainty: 38–48%), the frequency of *No* conflict and *No* uncertainty was relatively high in JOY, PRIDE and SADNESS (range 35–55%, depending on event and constellation). For instance, *No* answers were most frequent for JOY (respectively 48% and 45%) and PRIDE (respectively 55% and 44%).

Although a few participants simply said ‘*There is no reason to feel uncertain’* (or ... ‘*in conflict’*), most explained their No answer (see Table A4, bottom sections in the Supplementary Materials). Beliefs were a specular reflection of the reported causes for conflict or uncertainty presence. Namely, the absence of either phenomenon was believed to occur when SP does not feel opposite, ambivalent emotions (Q100–101), and/or does not feel unclear emotions—e.g., because SP is aware of her emotions and/or accepts them (Q102). Emotion awareness, for participants, is due to strong intensity of emotions, and/or clarity about their cause (Q109–112); however, feeling clear emotions does not imply lack of either intra-psychic or expression regulation (Q104–106). Emotions are clear also when they are all pleasant ones (Q107–110). Finally, some beliefs focused on event-specific reasons, such as the clarity or certainty of SP’s event appraisal (Q113–117), the event implications for SP’s goals (Q113) or the future (Q117). In sum, the conditions for *not* experiencing either phenomenon include clarity of felt emotions (and appraisals, action tendencies, etc.), due to their intensity, uniqueness or pervasiveness, SP’s ability to reflect upon her experience, and a ‘reason–emotion’ congruity. The latter cause further supports the hypothesis that, in participants’ beliefs, checks on emotion-norm conformity explain how people react to their own emotional experience and whether they will find it necessary to regulate it. Participants, as reported, drew in fact a distinction between knowing what one feels and *pretending* that one does not know, or *letting others believe that one does not know*: accordingly, rather than unclear felt emotions, sometimes SP is simply unwilling to accept her emotions, or let others know about them. Thus, even when participants believe that felt emotions do not imply either conflict or uncertainty, regulation is still salient. Overall, beliefs explaining the *absence* of either conflict or uncertainty contribute to confirm all stated hypotheses.

## Discussion

The aim of this paper was to study beliefs about conflict and uncertainty over felt emotions, and to assess whether they varied according to constellation valence, nature of eliciting events and of felt emotions. Conflict and uncertainty attributions were expected to vary as a function of emotion hedonic tone and norm-congruency (and norm clarity) from a personal and/or social viewpoint in relation to a specific event (context), and the presence of complex, mixed, and ambivalent felt emotions. Attributions were expected to involve a rich set of beliefs, many of which implicitly or explicitly would refer to regulation processes. The results reported in this paper were obtained in parallel studies conducted on the emotion constellations JOY, PRIDE, SADNESS, JEALOUSY and ENVY.

The reported quantitative and qualitative study results (see Tables 1 and 2, and the Sections Beliefs About Conflict and Uncertainty: Yes/No Attributions and Beliefs About the Causes of Conflict and Uncertainty) showed that conflict and uncertainty over felt emotions are *believed* to be frequent, almost inherent to emotional experiences because of their complexity and/or because some dysphoric element is present even when the overall hedonic tone is positive, and/or because one or more experience aspects are discrepant with respect to (idiosyncratic, or socially shared) emotional norms. The results, overall, supported the general hypotheses of the study, i.e., conflict and uncertainty attributions over felt emotions are more likely when the overall hedonic tone of the experience is unpleasant, for ‘serious’ than ‘light’ events, due to the greater salience of the former, when a felt *emotion, and/or its expression,* is perceived as norm-incongruent, and when the experience includes concomitant and/or ambivalent emotions. Expressed beliefs on the whole imply a malleability theory of emotions, and emotion awareness, thus supporting recent results (Ford & Gross, 2019). The results also supported further specific hypotheses on uncertainty (see Hypotheses section), i.e., it is greater when the experience is complex, including concomitant emotions (of a similar or opposite hedonic tone), making it difficult to grasp their cause, implications, etc., and when the person is uncertain about the need to regulate, and how, felt emotions.

The results, overall, showed that both conflict and uncertainty attributions to story protagonist SP are based on a large variety of beliefs (many of which focused on event appraisal and implications), with a common (usually implicit) basis, namely that emotional experiences often need to be regulated to maximize the experiencer’s intra-psychic well-being as well as her ‘social adequacy’—although when several goals are active at the same time it is difficult to manage conflict/uncertainty over which goal to pursue. More specifically, for both phenomena and all constellations, beliefs frequently focused on event novelty and other *appraisal dimensions*, including agency (especially intentionality), norm congruency, relevance for the self (self-concept, and personal goals, concerns), coping potential, and implications for the future. Many beliefs focused on action tendencies and, again, on their adequacy with respect to SP’s goals, concerns, self-concept, as well as SP’s relationship—at present and in the future—with other people involved in the event. In relation to appraisals and related action tendencies, in most beliefs regulation issues were implicit (sometimes explicitly mentioned), especially when focusing on the ‘most adequate’ event evaluation and its implications. Furthermore, beliefs quite often explicitly focused on other emotion components, especially emotion hedonic tone and intensity, and, less often, emotion duration, and on the complexity of emotional experiences, highlighting issues that become salient when feeling concomitant emotions, e.g., the need to evaluate their significance, or the most appropriate appraisal or action tendency among those associated with the concomitant emotions.

In sum, participants focused on *specific features and components* of emotions, including hedonic tone, intensity, action tendencies, event appraisal and implications (e.g., for SP’s self-concept, or the future), and on basic aspects of the emotion process, such as emotion regulation. For instance, when referring to concomitant emotions (e.g., anger and anguish; sadness and relief), conflict and/or uncertainty were often explained in terms of emotion-related differences—in hedonic tone, action tendencies, event appraisals, implications for self-concept, norm-congruence, etc.

The *content* of emotional-phenomena beliefs, their repertoire *richness* and *articulation* level however *differed across individuals*—who, for a given event, differed in the expected elicited emotions (e.g., envy rather than anger), or the emotional standards adhered to (e.g., feeling envious as un/comfortable), or which conclusion was drawn given the ‘same information’—e.g., an intense emotion, or a specific emotion, elicited uncertainty for some, but was the very cause for its absence for others. The major individual differences that were observed on the basis of the explanations (beliefs) collected in this study might be summarized as follows.

First, participants differed in number of expressed beliefs, their explanation *complexity*. Quite a few simply answered ‘*The event is unexpected’* or *‘… has many consequences’* as a reason for both phenomena, or provided generic (but not unreasonable) causes such as ‘*If he knew why he was uncertain, he would not be uncertain*!’ or ‘*Conflict is inevitable’*. Likewise, participants at times simply said ‘*No reason to be*....’ Most however explained either phenomenon supplying specific beliefs, not rarely complex ones, focusing on several issues (see Q5, Q6, Q15, Q16, Q30, Q51, Q58, Tables A1–A3 in the Supplementary Materials), highlighting more than one cause, such as Reason–Emotion plus Ambivalent emotions plus beliefs focusing on Implications for self-concept.

Defining *individual belief complexity* or *richness* is, however, neither a theoretically straightforward task nor an easy one. As the quotes illustrate (Tables A1–A4 in the Supplementary Materials; see also the section, 'Beliefs About the Causes of Conflict and Uncertainty'), we might hypothesize that each participant *P* focused on what—the belief(s)—was immediately most salient to her as a potential explanation. That is, the greater or lesser complexity in accounts of either phenomenon does not imply that *P*’s expressed beliefs are the only ones that for *P* explain the phenomena, nor that *P* has a poor belief repertoire, i.e., a repertoire in which other relevant beliefs are absent. Second, participants differed in whether they supplied distinct explanations for conflict and uncertainty: answering the uncertainty question, that *followed* the conflict one, some just replied, ‘*Same reasons’* or, ‘*As said above’*. In such cases it is impossible to know if participants actually believed uncertainty could be explained by the cause(s) they invoked for conflict (e.g., ‘*The event is unexpected’*), or if they were just tired, or unmotivated to answer (Zammuner, 2023, N5). In sum, further studies, perhaps relying on both closed- and open-question methods, are necessary to assess individual differences more precisely.

As a *collective* entity, people's beliefs were quite varied—especially in relation to differences between events and constellations - and complex, as quotes illustrate (Tables A1–A4 in the Supplementary Materials). The reported qualitative results on the whole confirm that people possess rich lay theories of emotions, comprising beliefs that might in turn focus on this or that component of the emotion response (e.g., typical behavioral and expressive responses, norms), as well as on aspects of major emotion processes (e.g., regulation) and their causes, in relation to emotion-specific antecedents. The results thus support the very general hypothesis (see the section, 'Lay Emotion Theories as Influences on Emotion Experience and Its Judgement') that people have an in-depth knowledge of emotions (lay theory) and rely on it when understanding and evaluating how someone reacts to an emotion-inducing event, why it so happens, and to what extent her emotional response and behavior is appropriate, normative—study participants did identify with the story protagonist, putting themselves in his/her shoes, as quotes indicate (Tables A1–A4 in the Supplementary Materials). We might argue that, thanks to the many opportunities to get to know how, why, and when ourselves or others experience this or that emotion (e.g., when sharing emotions, reading books, seeing a movie, etc.), such emotional empathy and cognitive perspective taking are, as it were, routine tasks throughout one's life span. Because emotions by their very nature are subjectively salient processes, people are motivated to continuously expand and update, as it were, their emotion knowledge, possibly checking whether it is congruent with significant others', with one's community and culture too. The results also showed that conflict and uncertainty beliefs include descriptive and prescriptive beliefs too (see the section, 'Lay Emotion Theories as Influences on Emotion Experience and Its Judgement') both of a superordinate nature (e.g., felt emotions might cause conflict (uncertainty) over their nature, their appropriateness), as well as more specific beliefs, i.e., emotion- and context-specific beliefs that differ across emotion constellations and events.

In conclusion, the reported study results on lay beliefs on conflict and uncertainty over felt emotions contribute, by expanding on related literature findings (see the section, 'The Study of Beliefs About Emotions'), to our understanding of naïve, lay theories of emotion as socially-based representations comprising, at their core, beliefs about the complexity of emotions (and their components), and the extent to which experiences are norm-congruent as well as functional to achieve concerns and goals made salient by the eliciting event, including those dictated by actual or ideal self-concepts. The results overall indicate that, in lay thinking, event protagonists try to modulate, regulate their emotional experiences and associated behaviors by taking into account what the transactional context affords, or *vice versa* prohibits. Future studies might further investigate what aspects of naïve theories of emotion are culture-specific, and to what extent. The study indirectly supports the hypothesis that beliefs about emotion influence people’s understanding of, and reactions to, their own emotions, and of how others are expected to understand, and react to, such experiences, and therefore affect the unfolding of the emotion process. To really understand a person’s emotional transactions with the world, we thus need to know her lay theory, because the latter influences the former (as much as the former influences the latter). In sum, the relevance of this special kind of naïve theory, namely naïve theories of emotion, might be more readily acknowledged than it has been so far.

## Supplementary Materials

The supplementary materials provided are the notes, tables, and associated references that support the findings of this study (for access see Index of Supplementary Materials below).



ZammunerV. L.
 (2023). Supplementary materials to "Naïve theories of emotions: Why people might (not) be uncertain or in conflict about felt emotions"
[Notes, tables, references]. PsychOpen. 10.23668/psycharchives.12871
PMC1050820837731896

## References

[sp1_r1] Zammuner V. L. (2023). Supplementary materials to "Naïve theories of emotions: Why people might (not) be uncertain or in conflict about felt emotions" [Notes, tables, references]. PsychOpen. 10.23668/psycharchives.12871 PMC10508208 37731896

